# PCTC-Net: A Crack Segmentation Network with Parallel Dual Encoder Network Fusing Pre-Conv-Based Transformers and Convolutional Neural Networks

**DOI:** 10.3390/s24051467

**Published:** 2024-02-24

**Authors:** Ji-Hwan Moon, Gyuho Choi, Yu-Hwan Kim, Won-Yeol Kim

**Affiliations:** 1 Department of Artificial Intelligence Engineering, Chosun University, Gwangju 61452, Republic of Korea; moonjh16@chosun.kr (J.-H.M.); ghchoi@chosun.ac.kr (G.C.); 2 Department of Computer Engineering, Chosun University, Gwangju 61452, Republic of Korea; asm6788@chosun.ac.kr

**Keywords:** crack, segmentation, CNN, transformer, PCTC-Net, Pre-Conv

## Abstract

Cracks are common defects that occur on the surfaces of objects and structures. Crack detection is a critical maintenance task that traditionally requires manual labor. Large-scale manual inspections are expensive. Research has been conducted to replace expensive human labor with cheaper computing resources. Recently, crack segmentation based on convolutional neural networks (CNNs) and transformers has been actively investigated for local and global information. However, the transformer is data-intensive owing to its weak inductive bias. Existing labeled datasets for crack segmentation are relatively small. Additionally, a limited amount of fine-grained crack data is available. To address this data-intensive problem, we propose a parallel dual encoder network fusing Pre-Conv-based Transformers and convolutional neural networks (PCTC-Net). The Pre-Conv module automatically optimizes each color channel with a small spatial kernel before the input of the transformer. The proposed model, PCTC-Net, was tested with the DeepCrack, Crack500, and Crackseg9k datasets. The experimental results showed that our model achieved higher generalization performance, stability, and F1 scores than the SOTA model DTrC-Net.

## 1. Introduction

Cracks in materials such as asphalt, concrete, and metals are of significant interest in many industrial fields. Fine-grained cracks not detected early can develop into coarse-grained cracks. These coarse-grained cracks result in serious human casualties and functional losses in areas like roads, buildings, ships, and aircraft. Particularly, asphalt and concrete, which are the primary materials for roads, are highly vulnerable to rain and moisture, making it difficult to completely prevent cracking. Therefore, early detection of minor cracks in paved roads is crucial for preventing human casualties and enhancing durability. Crack detection methods are classified as human visual inspections and computer vision.

Human visual inspection is variable in cost, time consumption, and reliability because it depends on the operator. It cannot ensure consistently reliable quality for industrial applications [1]. Earlier studies on the replacement of human resources with reliable computing resources were based on computer vision algorithms [2]. However, these algorithms also depend on experience and are not only inherently ambiguous but also highly specialized for specific cracks [3], as introduced by Koch et al. [4], Spencer et al. [5], Ye et al. [6], and Hu et al. [7]. Owing to these limitations, end-to-end deep-learning-based computer vision is advancing.

In deep learning, the processing of crack images evolves into two stages: object detection and segmentation. Object detection enables the detection of objects by delineating their boundaries using bounding boxes and classifying targets. For example, faster R-CNNs, YOLO, MobileNet, and SDDNet, etc. are generally lightweight and can quickly infer information from cracks [8,9,10,11]. In the field of crack detection, the challenge lies in the lack of standardized definitions for cracks, unlike more easily identifiable objects such as humans or vehicles, which affect the performance of detection methods.

Accordingly, recent studies have focused on segmentation models that can detect cracks at the pixel level. Convolutional neural networks (CNNs) are widely used in crack segmentation to extract local information. However, the convolution layer is constrained by the fixed receptive fields. Therefore, multiscale crack detection is challenging for small-scale CNN. To detect multiscale segmentation, effectively stacked CNN models (Fully Convolutional Networks (FCN) [12], Unet [13], and SegNet [14]), such as encoder-decoder and skip connections, have been researched. These models also demonstrate acceptable performance in crack segmentation. However, the challenge of expanding receptive fields remains a significant obstacle to effectively extracting global information from intricate images of cracks [15].

Transformers are a promising method for extracting global information to address receptive field limitations. Incorporating a transformer with its independent attention mechanism as a distinct encoder allows the utilization of global features unique to transformers [15]. Simultaneously, it maintains the local information that is intrinsic to a CNN encoder. SegFormer [16] and DTrC-Net [17] showed that the use of transformers significantly improved global information capture. Most segmentation research, including cracks, relies on high-quality, large-scale public datasets. However, it is difficult for the transformer to learn from a small dataset because of its weak inductive bias. Inductive bias is a set of assumptions inherent in the model architecture. CNNs have strong inductive biases such as translation invariance and locality [18]. However, transformers do not have strong inductive biases like CNNs, which is why transformers learn inductive biases implicitly from large amounts of data [19]. However, the currently available public crack datasets are relatively small [20]. Moreover, these public datasets are not suitable for learning fine-grained cracks. The fine-grained images are small and easily confused with the background, leading to their exclusion from datasets owing to perceived low quality or non-detection amidst numerous images. Constructing a large labeled dataset of fine-grained cracks requires significant time and cost. It is crucial to enforce learning from available fine-grained crack data in public datasets. However, due to the low inductive bias of transformers, they require large datasets, making it difficult to learn fine cracks. Therefore, research is needed to improve the inductive bias of transformers.

In this study, we propose a parallel dual encoder model called Parallel Dual Encoder Network (PCTC-Net), which is a fusion of Pre-Conv based Transformers and convolutional neural networks to improve the inductive bias of transformers. The Pre-Conv module automatically optimizes each color channel with small spatial kernels before transformer input to enhance the transformer’s inductive bias.

## 2. Related Work

### 2.1. CNN Models

The traditional segmentation methods divide the entire image into overlapped square image patches [21,22]. The patches are individually input into the classifier CNNs. For example, the Sliding Window Convolutional Neural Network (SW-CNN) [23] applies the convolutional operation at multiple locations across the input image. However, patch-based approaches must apply convolution operations to many patches. It occurs as a computational bottleneck [22].

Fully Convolutional Networks (FCN) is the first end-to-end segmentation model that does not use a fully connected layer, and all layers are composed of CNNs only. FCN uses the entire image instead of patches to annotate at once. FCN reduced computational bottlenecks and showed good accuracy compared to traditional patch-based approaches. The work of Katsamenis et al. [22] that segmented rust on metal in construction infrastructure demonstrated processing time per image proximately 102 times and a lower F1-Score than annotating the entire image in a one-pass architecture, FCN. However, because of the fixed receptive field, the prediction of small and large objects is inaccurate. DeepCrack [24] suggests an expansion of FCN by applying deeply supervised nets (DSN) [25] to utilize both low and high features. DSN is used for direct supervision to train each convolutional layer effectively. By using DSN, the network can effectively utilize features of various scales at each convolution layer, potentially improving its performance. However, the loss of spatial information and the resulting lower resolution make its application in precise crack segmentation challenging.

U-Net was originally a successful model for medical image segmentation and was naturally applied to crack segmentation because medical images and crack segmentation share many similarities, such as large backgrounds and small targets. The work of Hou et al. [26] is a prime example of early research that utilized U-Net to directly infer crack segmentation from crack images. SegNet has a simple structure and efficient parameter reduction using positional information inferred in the encoding and upsampling phases.

R2AU-Net [27] combined attention recurrent residual and standard U-net architecture. The main contribution of R2AU-Net is that it uses the user’s interaction to train the network. It performed higher scores than other models based on U-Net, but because it is semi-supervised learning, it requires user intervention, so end-to-end crack learning is not possible.

However, even when utilizing various scales, CNN models still face limitations because of their inherent architectural characteristics, which make it challenging to expand local receptive fields, thereby hindering the capture of global information [15].

### 2.2. CNN and Transformer Fusion Models

The transformer is a deep learning structure comprising embeddings and self-attention [28]. The transformer demonstrated excellent performance in extracting global information for natural language processing [29]. Similarly, the global information-extraction capacity of transformers is useful in computer vision. The Vision Transformer (ViT) was the first to use transforms for computer vision, but it is difficult to extract fine spatial information because the number of tokens and their dimensions are fixed [30]. The SegFormer [16] stacks the transformers into a Multi-Scale without a CNN. The global information extracted for each layer led to high performance. However, it cannot extract local information. To address the limitations in capturing local information, combining CNN demonstrates improved performance compared with merely increasing the transformer layers [31]. For example, SegCrack [20] proposed a method using a pyramid structure combining transformers and CNNs to extract both the local and global features of cracks. SegCrack reduces the sequence length using a reduction factor to lower the computational complexity. However, shortening the sequence length results in the loss of the original information.

TransUNet [32] has recently been applied to the study of crack segmentation [33]. TransUNet inputs features extracted from the CNN layer (ResNet-50) into the transformer and utilizes them in the decoder via skip-connection. Although it improves performance, it is not suitable for fine-grained crack segmentation because it blurs fine-grained cracks.

FAT-Net [34] was originally used in skin lesion segmentation. Similar to U-Net, it was applied to crack segmentation due to the similarities between medical imaging and crack segmentation. FAT-Net uses a CNN and transformer dual-encoder methodology. FAT-Net’s dual encoders are independent of each other until the outputs of the CNN encoder and transformer encoder are combined. Therefore, FAT-Net lacks the propagation of global information.

DTrC-Net also uses a CNN and transformer dual-encoder methodology, but fuses many of the features of the CNN encoder and transformer. It consists of a transformer encoder, CNN encoder, feature fusion module (FFM) module, RPM module, and decoder. The FFM was designed to improve the fusion of the information extracted from the two encoders. The residual path module (RPM) is designed to improve the semantic difference between the encoder and decoder. The RPM automatically optimizes the distribution of the feature map for the effectiveness of the training process. However, models such as DTrC-Net and SegCrack primarily focus on the fusion of encoders and decoders and pay less attention to the transformer encoder itself. This leads to the weakening of the inductive bias of the transformer encoder and an overall contextual understanding, which is critical in the field of crack segmentation, where datasets are often not extensive. Therefore, it is necessary to strengthen the inductive bias of the transformer encoder specifically for crack segmentation.

### 2.3. Loss Function

Crack segmentation poses a significant challenge owing to the pronounced class imbalance in crack data [35]. The dataset exhibits an overwhelming prevalence of background pixels, vastly outnumbering the relatively scarce pixels representing cracks. Therefore, it is important to design a loss function that can solve the problem of dataset imbalance. There are two types of losses: those caused by the aforementioned characteristics and those caused by the segmentation itself.

Segmentation losses can be categorized into four types: distribution-based, region-based, boundary-based, and compounded [36]. In this study, we focused only on the common cross-entropy and distribution-based loss functions and the combo loss used in this experiment. Distribution-based loss is a loss classification that modifies positive and negative weights based on cross-entropy [37]. Since it is calculated for each pixel, it has the problem that the background affects the crack more than the crack, so additional weights are needed to tackle the crack [38,39]. However, the convergence was fast, and the loss was stable.

The region-based loss is calculated by directly transforming a metric into a loss to maximize a metric that measures the similarity between two samples. An example is dice loss [40]. The disadvantage is that even a small difference from the correct answer has the same effect as missing a large object in distribution-based loss; therefore, learning is unstable and takes longer to converge. To solve this problem, combo loss [41] has been proposed, which is generally a combination of distribution- and region-based methods. Typically, it combines cross-entropy with ice. It is useful for solving the class imbalance problem because it can utilize both the flexibility of the dice loss and the stability of the cross-entropy loss. Therefore, in this study, we designed a suitable combination loss for the proposed PCTC-Net through experimental verification.

## 3. Methodology

### 3.1. PCTC-Net

**Figure 1:**
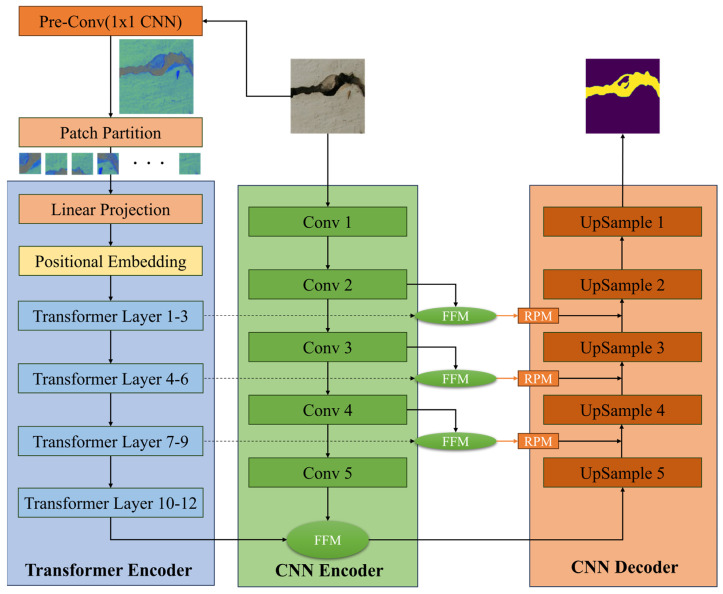
Overview of the proposed method of PCTC-Net.

In this study, we proposed the parallel dual encoder network fusing Pre-Conv-based Transformers and convolutional neural networks (PCTC-Net). Figure 1 shows the overall structure of the proposed PCTC-Net. The structure of PCTC-Net is based on that of DTrC-Net, which comprises an encoder and a decoder. The encoder is a dual-encoder network that fuses a convolutional neural with a transformer that incorporates our Pre-Conv module. The transformer encoder of PCTC-Net was designed to extract the global features of the crack images. First, the raw image is input to the Pre-Conv module to strengthen the inductive bias of the transformer. Next, the feature image output from the Pre-Conv module is divided into patches to be fed to the transformer encoder. The size of the patch is 16 × 16 without overlapping. Since the patched image is a 2D patch, flattening is performed in one dimension. Then, the patches flattened into a 1D array are linearly projected into a sequence of tokens. Finally, positional embedding is added to the patch tokens to maintain the relative position of the patches. The formula for the positional embedding (PE) operation [28] is as follows:

(1) $${PE}_{\left(pos,2i\right)}=\sin\left(\frac{pos}{{10,000}^{\frac{2i}{d}}}\right)$$

(2) $${PE}_{\left(pos,2i+1\right)}=\cos\left(\frac{pos}{{10,000}^{\frac{2i}{d}}}\right)$$

pos represents the position where the sequence length is located, d represents the dimension of the vector, and i represents the feature dimension corresponding to each sequence. For example, the sin at even positions and the cos at odd positions for features. Each transformer layer contained Multi-Head Attention (MHA). MHA is a variant of the standard attention mechanism that calculates attention scores using Q, K, V matrices in parallel. These Q, K, V represents Query, Key, Value, respectively. MHA transforms Q, K, V transforms linear projections.

(3) $$Q,\;K,V=QW^{Q},\;KW^{K},VW^{V}$$

where $W^{Q}$, $W^{K}$, $W^{V}$ denote parameter matrices. These matrices of weights are optimized to obtained the suitable weights to fit the true values. The Attention Score is calculated based on the following formula:

(4) $$Attention\;\left(Q,K,V\right)=\mathrm{softmax}(\frac{Q\cdot K^{T}}{\sqrt{d_{k}}})$$

The value $d_{k}$ calculated as $\frac{d}{h}$, where h is the number of heads in the Multi-Head Attention (MHA). The MHA calculations based on the following formula:

(5) $$MultiHead\;\left(Q,K,\;V\right)=Conact\left({head}_{1},\;\ldots ,\;{head}_{h}\right)W^{0}\;where\;{head}_{i}=Attention(QW_{i}^{Q},\;KW_{i}^{K},VW_{i}^{V})$$

The matrices $W_{i}^{Q},W_{i}^{K},W_{i}^{V}\;$ represent the projections of Q, K, V onto the *i*-th subspace. $W^{0}$ is the matrix used for the linear transformation of the attention head. The CNN encoder was designed based on ResNet [42] to ignore the background and extract the local features of the cracks. To mitigate the vanishing-gradient problem, the encoder uses a shortcut with a residual block. The residual block had multiple convolutions, batch normalization, and ReLU layers. The first module had single convolution, batch normalization, and ReLU layers. The other modules had three, four, six, and three residual blocks, respectively. Two submodules were used in the PCTC-Net. First, a feature fusion module (FFM) was designed for adaptive weight assignment. The fusion features of the CNN and transformer are also important for preventing information loss and maintaining local information. Therefore, we do not only fuse the outputs of the final layer. Second, the residual path module (RPM) is designed to reduce the semantic gap between the encoders. The semantic gap refers to the disparity between low-level image features captured by algorithms and high-level semantic concepts (keywords and categories) interpreted by humans. The decoder was designed to reach the target resolution by upsampling the layers. The input of the first upsampling layer fuses the feature maps from the two parallel encoders via the FFM module. The other layer uses a fusion feature map from each FFM, RPM, and the previous layer. Subsequently, the information processed through the fusion modules for the features extracted from the two encoders is hierarchically stacked as an additional input in each upsampling module. The proposed model is modified by focusing on the transformer layer. Maximize the learning of the global features of crack images to capture fine-grained cracks.

### 3.2. Pre-Conv Module

The original transformer module used in the encoder receives the input in the form of raw or patch data. However, the Transformer has a weak inductive bias and requires a substantial amount of data for effective learning. Obtaining a large, labeled dataset is challenging because of crack characteristics. Insufficient data can lead to overfitting, hindering the learning process. This weak inductive bias makes it difficult to make the model lightweight. A reduction in the number of transformer layers also led to a reduction in the number of parameters. This makes the already difficult training even more difficult. Therefore, we required light layers to compensate for the reduced number of layers.

However, ViT typically employs convolution with an equal kernel size and stride of 1 × 16 × 16 when patching an input image. The patching operation did not inject any information into the transformer. In this study, we propose a visual processing method suitable for crack segmentation.

**Figure 2:**
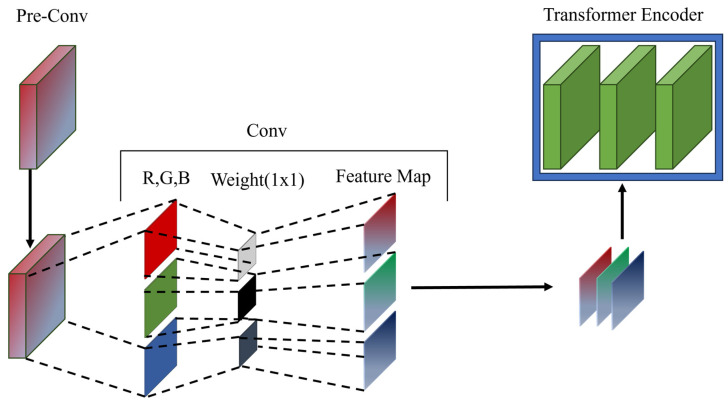
Schematic of the proposed Pre-Conv module.

Our “Pre-Conv” is an alternative approach to raw image patches for injecting inductive bias into transformers. Figure 2 shows a schematic of the proposed Pre-Conv module. Pre-Conv is a stem color emphasis layer that maximizes the color difference. It is purposefully simple and is not designed to maximize model accuracy. The Pre-Conv module is a single 1 × 1 kernel CNN layer. The Pre-Conv operates sequentially. Initially, convolutional operations are applied to each color channel (R, G, and B) of the input image using the respective 1 × 1 kernels. Subsequently, the resulting feature maps are amalgamated by transforming them back into a unified image, which is fed into the transformer for further processing.

Generally, deep CNN approaches extract high-level local feature maps using more than one kernel. However, the convolution operations blur the pixels. Blurred pixels are a critical disadvantage in crack segmentation, and the color transition of fine-grained cracks is important. Furthermore, high-level local feature maps cause a local bias in the representation of the transformer. CNNs enhance the local inductive bias, but transformers require enhancement of the global inductive bias for the effective segmentation of fine-grained cracks. A single-layer 1 × 1 kernel CNN approach is advantageous for injecting a global inductive bias into a transformer. Additionally, the use of Pre-Conv is advantageous because it employs a kernel size of 1, eliminating the need for square operations and thereby significantly reducing the computational load.

## 4. Dataset and Experiment Environment

We used the public datasets DeepCrack [24] and Crack500 [43] to evaluate the performance of PCTC-Net in detecting cracks in paved roads made of asphalt and concrete. We also utilized the public dataset Crackseg9k [44] to evaluate whether PCTC-Net performs well in detecting cracks in a variety of materials, including ceramic, glass, and masonry. The dataset sizes were 539 for DeepCrack, 3020 for Crack500, and 9255 for Crackseg9k. The DeepCrack dataset contains 539 images depicting cracks of various pixel sizes, with 300 images designated for training and 239 images for testing. This dataset encompasses diverse road crack images with a notable presence of smooth background data. The DeepCrack dataset enables the learning of features, such as crack length and width, through a comprehensive set of data. The Crack500 dataset comprises 500 images of road cracks captured using mobile phones at a resolution of 2000 × 1500 pixels at Temple University. Through cropping, the dataset was divided into 1896 training and 1124 testing images for the analysis. Crack500 has a high resolution and a substantial amount of rough background data, enabling the learning of detailed features of road cracks. The Crackseg9K dataset comprises 9255 diverse crack images, adjusted to a resolution of 400 × 400 pixels. This dataset is a composite of ten datasets, including Sdnet, Cracktree, and Ceramic, and encompasses various types of cracks. The DeepCrack dataset exhibited an average crack coverage of approximately 3.58% per image, whereas the Crack500 dataset showed a percentage of 6.03% and the Crackseg9k dataset comprised 5.09%. To evaluate the performance of the proposed model, it was implemented on three datasets: DeepCrack, Crack 500, and Crackseg9k. The experiments were conducted using an RTX 4090. For each dataset, the input images were standardized to 256 × 256 pixels.

Applying data augmentation to mitigate overfitting in the learning model, a combination of transformations, including Flip, Rotate, HorizontalFlip, and RandomRotate90 with a ratio of 0.7, a variable ratio of VerticalFlip, brightness contrast with a ratio of 0.3, and random gamma with a ratio of 0.5, were employed. The optimization utilized AdamW, with a loss function comprising 0.5 dice and 0.5 focal loss. The model was trained with a batch size of 3, over 300 epochs, and StepLR with an initial learning rate of 0.0001 and a gamma of 0.1. The segmentation performance of the proposed model was quantitatively evaluated using precision, recall, and F1-score as the assessment metrics.

Precision represents the proportion of predicted cracks that are actually cracks, and a higher precision indicates fewer false positives (*FP*). Precision is defined as follows:

(6) $$Precision=\frac{TP}{TP+FP}$$

The recall denotes the proportion of actual cracks that were correctly predicted, and a higher recall indicates fewer false negatives (*FN*). Recall is defined as

(7) $$Recall=\frac{TP}{TP+FN}$$

The *F*1-score serves as the harmonic mean of precision and recall, effectively capturing the importance of both metrics when crucial. The *F*1-score is defined as

(8) $$F1\;score=\frac{2\times Precision\times Recall}{Preicision+Recall}$$

Precision, recall, and *F*1-score serve as metrics for evaluating and optimizing the performance of a model by assessing how effectively it classifies pixels.

To analyze the optimized loss function for the proposed PCTC-Net, the experimental environment was configured with three types of loss functions: dice loss, BCE loss, and focal loss. The dice, BCE, and focal losses were calculated as follows:

(9) $$DiceLoss\left(P,G\right)=1-\frac{2\times \left|P\cap G\right|+smooth}{\left|P\right|+\left|G\right|+smooth}$$

(10) $$BCE(P,G)=-[G^{\prime }\log\left(s\left(P\right)\right)+\left(1-G\right)\log\left(1-s\left(P\right)\right)]$$

(11) $$FL\left(p_{t}\right)=-\alpha_{t}{\left(1-p_{t}\right)}^{r}log(p_{t})$$

P and G in each formula represent pred and ground truth, respectively. In dice loss, smoothing is set to 1, and in BCE loss, it refers to the sigmoid function. For focal loss, r was set to 0.25, and $P_{t}$ is the model’s prediction probability for a crack image.

## 5. Experimental Results

In this study, to determine the optimal parameters for the proposed PCTC-Net, experiments were conducted to compare the kernel size of Pre-Conv, batch size, and loss function. Furthermore, to evaluate the potential of model lighting, experiments were performed to reduce the number of transformer layers. In addition, to verify the flexibility of the model, comparative experiments with the DTrC-Net were conducted using three datasets: DeepCrack, Crack500, and Crackseg9k. In the experiments involving the Pre-Conv kernel size, batch size, learning rate, loss function, and number of transformer layers, a 1 × 1 Pre-Conv, batch size of 3, and loss function of 0.5 dice + 0.5 focal were used, and the learning rate was set using StepLR with an initial value of 0.0001. For the experiments to verify the model’s flexibility, the DTrC-Net’s baseline settings were a batch size of 16, a loss function of 0.75Dice + 0.25BCE, and the learning rate was the same as that of PCTC-Net.

Experiments were conducted based on the kernel size of Pre-Conv, and the results are presented in Table 1. The 1 × 1 kernel size exhibited the highest performance with an F1-Score of 87.31%, recall of 88.01%, and precision of 86.62%. 

Using Pre-Conv with 3 × 3 or 5 × 5 kernels can affect the performance of the Transformer Encoder, which is designed to extract global information because the inductive bias of the CNN plays a role in extracting local features. Additionally, there is a risk of losing the important features of the crack boundaries, leading to a decrease in performance. Therefore, a 1 × 1 convolution was selected as the kernel size for Pre-Conv to improve the inductive bias of the transformer.

The batch size influences the training time and model performance [45]. Therefore, to determine the optimal batch size, experiments were conducted with three different batch sizes (3, 5, and 10), and the results are presented in Table 2. In PCTC-Net, the highest performance was observed with an F1-score of 87.31% when the batch size was 3. However, an increase in batch size resulted in a decreasing trend. This trend is linked to the issue of crack segmentation, where there is a significant difference in the area between the crack and background in most images, causing a class imbalance. The batch size experimental results for both models indicate that a smaller batch size can positively impact specific datasets and environments. 

Owing to the severe class imbalance problem between the background and cracks in the crack dataset, a suitable loss function that can alleviate this issue is necessary. Therefore, to find the optimal loss function for our proposed model and the crack dataset, we conducted comparative experiments on five loss function combinations using three different loss functions (dice, BCE, and focal). The results are shown in Table 3. Analysis of the results trained with each loss function revealed that 0.75Dice + 0.25BCE achieved the highest performance with an F1-score of approximately 87.36%. However, in crack segmentation, recall is a more critical metric than precision from a safety perspective. As shown in Table 3, 0.5Dice + 0.5Focal, although approximately 0.05% lower in F1-score than 0.75Dice + 0.25BCE, was approximately 5.22% higher in recall. Therefore, the loss function for the proposed PCTC-Net was set to 0.5Dice + 0.5Focal.

**Figure 3:**
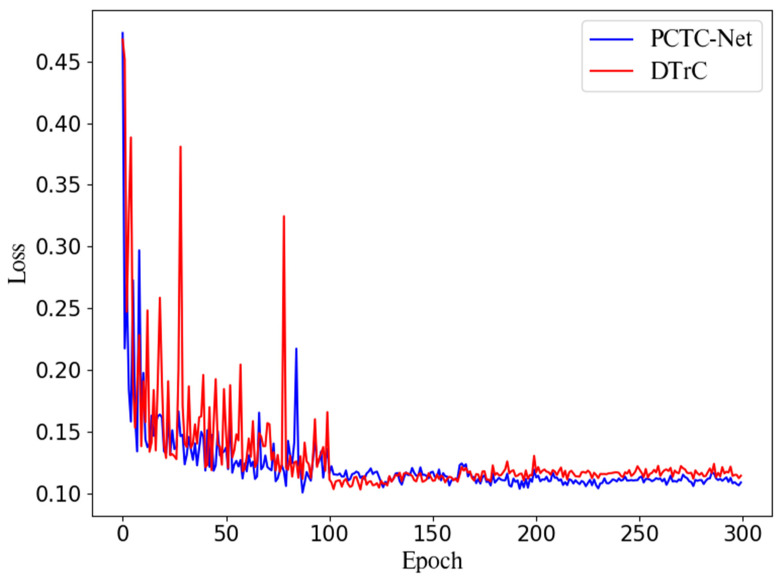
Comparison loss of PCTC-Net and DTrC-Net [17].

Experiments were conducted to verify the improvement in the inductive bias of the transformer and to examine the potential for model lighting. The results are shown in Table 4. As shown in Figure 3, each of the four blocks of the transformer contains three layers. Experiments were performed by reducing the number of transformer layers in each block by one, resulting in the use of 12, 8, and 4 layers. When the number of layers was reduced from 12 to 4, the DTrC-Net showed a performance decline of approximately 2.71% in terms of the F1-score, whereas the PCTC-Net only exhibited a decline of approximately 0.9%. This indicates that the Pre-Conv strengthens the inductive bias of the transformer and suggests its potential for model lighting. Moreover, PCTC-Net exhibited a performance difference of approximately 8.46% in recall compared with DTrC-Net. In addition to maintaining performance despite reducing transformers, it also made a significant contribution by increasing FPS from 215 to 244.

Experiments were conducted to compare the performance, computational cost, and timing between models with a single encoder structure and those with a dual-encoder structure, as illustrated in Table 5. In terms of accuracy, the dual-encoder structured PCTC-Net demonstrated the highest performance with an F1-score of approximately 87.31%. In contrast, the single-encoder-structured FCN and U-Net showed lower performances, with F1-scores of approximately 85.71% and 85.91%, respectively, when compared to the dual-encoder structures of PCTC-Net and FAT-Net. 

This suggests that the dual-encoder structure, utilizing both CNN and Transformer encoders, effectively combines global and local features to achieve superior performance. In terms of computational cost and timing, the dual-encoder structured PCTC-Net and FAT-Net did not exhibit superior model parameters and inference time compared to the single-encoder-structured models FCN and U-Net. However, as indicated in Table 4, the maintenance of performance despite a reduction in the transformer layers of PCTC-Net suggests the possibility of lightening the model and reducing inference time through fine-tuning of Pre-Conv and parameter combinations.

To observe the training convergence speed and stability of PCTC-Net, the change in the loss values at each epoch is visually represented, as shown in Figure 3. The training was divided into early, middle, and late stages, using every 100 epochs as a benchmark for comparison. When calculating the epochs in which PCTC-Net showed lower losses compared to DTrC-Net in each stage, it was found that in the early stage, PCTC-Net had better losses in 68 epochs, in the middle stage in 47 epochs, and in the late stage in 99 epochs. These results suggest that PCTC-Net demonstrated faster convergence in the early stages of training. Additionally, while PCTC-Net and DTrC-Net showed similar learning trends during the middle stage, PCTC-Net continued more stable training in the late stage. This suggests that the use of Pre-Conv enhanced the training convergence speed and stability of the transformer encoder. Therefore, it was demonstrated that the use of Pre-Conv contributed to the convergence speed and stable training of the model.

To verify whether the proposed PCTC-Net performs well in various scenarios and is not optimized for a specific dataset, experiments were conducted using the three aforementioned datasets. The performance of each model was evaluated using Precision, Recall, and F1-Score, and the results are listed in Table 6. The PCTC-Net model showed an average improvement of 0.82% in the F1-Score compared with the DTRC-Net model. In the DeepCrack dataset, PCTC-Net exhibited higher performance in terms of precision, recall, and F1-score. For the Crack500 dataset, PCTC-Net showed slight improvements in precision and F1-score of 1.24% and 0.31%, respectively. Finally, in the Crackseg9k dataset, PCTC-Net demonstrated slight improvements in all aspects. Consequently, the PCTC-Net model consistently showed better performance improvements across various datasets than the DTrC-Net.

**Figure 4:**
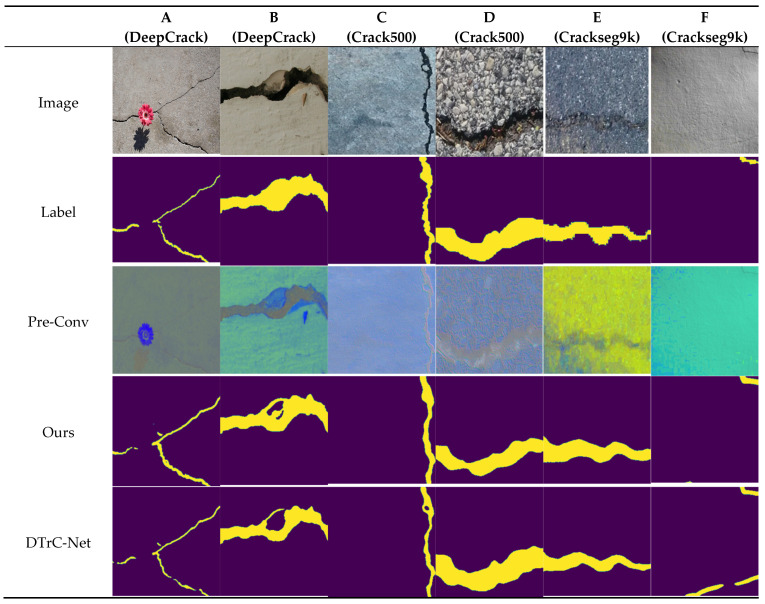
Crack prediction comparison results of PCTC-Net and DTrC-Net [17] on DeepCrack, Crack500, and Crackseg9k datasets.

Figure 4 illustrates the crack detection results for the six test images to visually compare the predictive performances of the PCTC-Net and DTrC-Net. Each set of six images was extracted from the DeepCrack, Crack500, and Crackseg9K datasets, with two images from each dataset.

As shown in Figure 4a, PCTC-Net represents cracks more accurately, whereas DTrC-Net tends to detect cracks incorrectly. Figure 4b categorizes the cracks into two types based on their brightness: bright and dark. The DTrC-Net failed to detect bright cracks accurately, whereas the PCTC-Net managed to detect some cracks. These observations suggest that the ability of the Pre-Conv layer to extract features from various types of cracks and feed them into the transformer leads to improved performance. Moreover, as shown in Figure 4c, the DTrC-Net failed to detect bright cracks, whereas the PCTC-Net successfully detected them.

In the image shown in Figure 4f, PCTC-Net demonstrates a better understanding of the characteristics of the cracks than DTrC-Net. Overall, PCTC-Net tended to detect cracks more accurately in various environments and conditions. However, the accuracy of the DTrC-Net for crack detection may be compromised under certain conditions. This comparison helps understand the strengths and weaknesses of each model and emphasizes the importance of utilizing them in appropriate environments.

## 6. Discussion

This paper proposes the PCTC-Net to improve the low Inductive bias in crack segmentation models using transformers. Additionally, to assess the performance of PCTC-Net, experiments were conducted to optimize four hyperparameters: pre-conv size, batch, loss function, and transformer layer. The performance was compared using three datasets (DeepCrack, Crack500, Crackseg9k) against the DTrC-Net.

The experimental results showed that the 1 × 1 size pre-conv had better performance than the 3 × 3 and 5 × 5 sizes. This is interpreted as CNN’s Inductive bias playing a role in extracting local features when using 3 × 3 or 5 × 5 size pre-conv, thereby affecting the performance of the transformer encoder. In the batch size experiment, the best performance was observed with a batch size of 3, with performance tending to decrease as batch size increased. However, the optimal batch size can vary depending on specific datasets and environments, so it should be carefully selected. For the loss function, 0.5Dice + 0.5Focal showed about 0.05% lower F1-score but about 5.22% higher Recall compared to 0.75Dice + 0.25BCE. Since the main objective of this study is to improve model performance for the enhancement of structural and human safety, 0.5Dice + 0.5Focal with higher Recall was adopted. Experiments conducted to examine the improvement of low Inductive bias in the transformer encoder and the possibility of model lightening showed that PCTC-Net, even after reducing the transformer layers from 12 to 4, only showed about 0.9% performance degradation. Additionally, FPS increased from 94 to 110. This is interpreted as the Pre-Conv improving the low Inductive bias of the Transformer encoder and also contributing to the model’s lightness. Observing the Loss Graph of PCTC-Net and DTrC-Net, PCTC-Net showed fewer loss peaks in the initial epochs and better loss convergence in the later epochs compared to DTrC-Net. Therefore, the use of Pre-Conv is interpreted as contributing to the improvement of the initial learning speed and later learning ability of the transformer encoder. In experiments conducted to compare PCTC-Net and DTrC-Net across various datasets, PCTC-Net showed good performance in all datasets, indicating consistent performance across different datasets. However, a drawback was that in the Crack500 dataset, PCTC-Net’s Recall was about 1.4% lower than DTrC-Net.

Overall, the Pre-Conv of PCTC-Net improved the low Inductive bias of the Transformer encoder, leading to enhanced performance. Generally, crack segmentation models using transformer encoders require large datasets, but the use of Pre-Conv contributed to mitigating this drawback. Especially, PCTC-Net has the significant advantage of not showing a substantial decrease in performance despite reducing the number of layers in the Transformer Encoder.

## 7. Conclusions

In this study, we proposed PCTC-Net to improve crack segmentation performance through the fusion of CNN and transformers. In experiments using three datasets, DeepCrack, Crack500, and Crackseg9k, PCTC-Net showed an average increase of 0.82% in the F1-Score compared to DTrC-Net. PCTC-Net was optimized by experimenting with the batch size, loss function, and kernel size of the Pre-Conv. When the transformer encoder is fed a Feature Map that has passed through CNN instead of raw images, the transformer can more effectively detect cracks and facilitate efficient learning. By feeding the Feature Map, which is enriched with pixel-level features through CNN, into the transformer encoder, the inductive bias of the transformer is enhanced. As seen in Figure 3, PCTC-Net demonstrates a better learning convergence speed compared to DTrC-Net. The results demonstrate that the proposed network architecture is highly suitable for crack segmentation using Pre-Conv instead of directly using image patches with transformers. PCTC-Net showed a minimal performance decline of only 0.9% in the F1-Score even when reducing the layers in the transformer blocks, whereas DTrC-Net showed a decline of 3.2%. This indicates that the Pre-Conv improves the inductive bias of the transformer and suggests its potential for model lighting. Our future plans focus on developing data augmentation and semi-supervised learning techniques for crack segmentation, aimed at maintaining robust performance against various types of cracks and complex backgrounds within a single image. We also plan to construct and test experimental datasets to evaluate crack segmentation performance under diverse conditions. Furthermore, we intend to conduct cross-domain research in medical imaging. This will expand the applicability of PCTC-Net to diverse environments beyond its limited conditions.

## Figures and Tables

**Table 1:** Pre-Conv size experiment results. The best performances for each metric across different Pre-Conv sizes are indicated in bold font.

Pre-Conv Size	Precision	Recall	F1-Score
X	85.46%	87.16%	86.11%
1 × 1	**86.62%**	**88.01%**	**87.31%**
3 × 3	85.48%	87.68%	86.57%
5 × 5	86.53%	87.59%	87.06%

**Table 2:** Comparison results of PCTC-Net and DTrC-Net [17] with different batch sizes. The best performances for each metric across different batch sizes are indicated in bold font.

Model	Batch Size	Precision (%)	Recall (%)	F1-Score (%)
PCTC-Net	3	**86.62**	88.01	**87.31**
	5	85.88	**88.03**	86.94
	10	85.33	86.30	85.81
DTrC-Net [17]	3	85.20	87.10	86.14
	5	85.08	87.82	86.43
	10	83.55	87.40	85.43

**Table 3:** Results of PCTC-Net trained with different loss functions. The best performances for each metric across different loss function are indicated in bold font.

Loss Function	Precision (%)	Recall (%)	F1-Score (%)
Dice	92.21	81.43	86.48
0.75Dice + 0.25BCE	**92.47**	82.79	**87.36**
0.5Dice + 0.5Focal	86.62	**88.01**	87.31
0.75Dice + 0.25Focal	86.10	87.48	86.78

**Table 4:** Comparison results of PCTC-Net and DTrC-Net [17] with different transformer layers. The best performances for each metric across different numbers transformer layers are indicated in bold font.

Model	Transformer Layer	Precision (%)	Recall (%)	F1-Score (%)	FPS
PCTC-Net	4	86.83	85.99%	86.41%	**244**
	8	86.81	86.82%	86.82%	224
	12	86.61	**88.03%**	**87.31%**	215
DTrC-Net [17]	4	**89.82**	78.96%	84.04%	244
	8	89.39	78.36%	83.51%	224
	12	87.23	86.27%	86.75%	215

**Table 5:** Average computational time per image for models with 1-path structure (FCN [12], U-Net [13]) and those with a 2-path structure(PCTC-Net, FAT-Net). The best performances for each metric across different model are indicated in bold font.

Type	Model	Precision (%)	Recall (%)	F1-Score (%)	Model Parameters (M)	Times (ms)
Single-encoder	FCN [12]	85.66	85.75	85.71	**13.334**	**1.113**
	U-Net [13]	**87.66**	84.24	85.91	31.037	2.728
Dual-encoder	PCTC-Net	86.61	**88.03**	**87.31**	66.162	4.642
	FAT-Net [35]	85.85	86.16	86.01	36.543	3.609

**Table 6:** Comparison results from DeepCrack, Crack500, and Crackseg9k using PCTC-Net and DTrC-Net [17].

Model	Dataset	Precision (%)	Recall (%)	F1-Score (%)
PCTC-Net	DeepCrack	86.62	88.01	87.31
	Crack500	64.92	85.83	73.92
	Crackseg9k	72.23	87.38	79.08
DTrC-Net [17]	DeepCrack	85.20	87.10	86.14
	Crack500	63.68	87.21	73.61
	Crackseg9k	71.98	85.33	78.09

## Data Availability

The data provided in this study are available from the corresponding author.

## References

[1] Medina R., Gómez-García-Bermejo J., Zalama E. (2010). Automated Visual Inspection of Road Surface Cracks. Proceedings of the 27th ISARC.

[2] Tang F., Han C., Ma T., Chen T., Jia Y. (2021). Quantitative analysis and visual presentation of segregation in asphalt mixture based on image processing and BIM. Autom. Construct..

[3] Wang Z., Xu G., Ding Y., Wu B., Lu G. (2020). A Vision-Based Active Learning Convolutional Neural Network Model for Concrete Surface Crack Detection. Adv. Struct. Eng..

[4] Koch C., Georgieva K., Kasireddy V., Akinci B., Fieguth P. (2015). A review on computer vision based defect detection and condition assessment of concrete and asphalt civil infrastructure. Adv. Eng. Inform..

[5] Spencer B.F., Hoskere V., Narazaki Y. (2019). Advances in Computer Vision-Based Civil Infrastructure Inspection and Monitoring. Engineering.

[6] Ye X.W., Jin T., Yun C.B. (2019). A review on deep learning based structural health monitoring of civil infrastructures. Smart Struct. Syst..

[7] Hu W., Wang W., Ai C., Wang J., Wang W., Meng X., Liu J., Tao H., Qiu S. (2021). Machine vision-based surface crack analysis for transportation infrastructure. Autom. Constr..

[8] Cha Y.-J., Choi W., Büyüköztürk O. (2017). Deep learning-based crack damage detection using convolutional neural networks. Comput. Aided Civ. Infrastruct. Eng..

[9] Alfarrarjeh A., Trivedi D., Kim S.H., Shahabi C. A Deep learning approach for road damage detection from smartphone images. Proceedings of the 2018 IEEE International Conference on Big Data (Big Data).

[10] Sandler M., Howard A., Zhu M., Zhmoginov A., Chen L.C. Mobilenetv2: Inverted residuals and linear bottlenecks. Proceedings of the IEEE Conference on Computer Vision and Pattern Recognition.

[11] Maeda H., Sekimoto Y., Seto T., Kashiyama T., Omata H. (2018). Road damage detection and classification using deep neural networks with smartphone images. Comput.-Aided Civ. Infrastruct. Eng..

[12] Long J., Shelhamer E., Darrell T. Fully Convolutional Networks for Semantic Segmentation. Proceedings of the IEEE Conference on Computer Vision and Pattern Recognition.

[13] Ronneberger O., Fischer P., Brox T. U-Net: Convolutional Networks for Biomedical Image Segmentation. Proceedings of the Medical Image Computing and Computer-Assisted Intervention–MICCAI 2015: 18th International Conference.

[14] Badrinarayanan V., Kendall A., Cipolla R. (2017). SegNet: A Deep Convolutional Encoder-Decoder Architecture for Image Segmentation. IEEE Trans. Pattern Anal. Mach. Intell..

[15] Zheng S., Lu J., Zhao H., Zhu X., Luo Z., Wang Y., Fu Y., Feng J., Xiang T., Torr P.H. Rethinking semantic segmentation from a sequence-to-sequence perspective with transformers. Proceedings of the IEEE/CVF Conference on Computer Vision and Pattern Recognition.

[16] Xie E., Wang W., Yu Z., Anandkumar A., Alvarez J.M., Luo P. (2021). SegFormer: Simple and Efficient Design for Semantic Segmentation with Transformers. Adv. Neural Inf. Process. Syst..

[17] Xiang C., Guo J., Cao R., Deng L. (2023). A Crack-Segmentation Algorithm Fusing Transformers and Convolutional Neural Networks for Complex. Autom. Constr..

[18] Han K., Wang Y., Chen H., Chen X., Guo J., Liu Z., Tang Y., Xiao A., Xu C., Xu Y. (2023). A Survey on Vision Transformer. IEEE Trans. Pattern Anal. Mach. Intell..

[19] Xu Y., Zhang Q., Zhang J., Tao D. (2021). Vitae: Vision transformer advanced by exploring intrinsic inductive bias. Adv. Neural Inf. Process. Syst..

[20] Wang W., Su C. (2022). Automatic Concrete Crack Segmentation Model Based on Transformer. Autom. Constr..

[21] Soukup D., Huber-Mörk R. (2014). Convolutional neural networks for steel surface defect detection from photometric stereo images. International Symposium on Visual Computing.

[22] Katsamenis I., Doulamis N., Doulamis A., Protopapadakis E., Voulodimos A. (2022). Simultaneous Precise Localization and Classification of metal rust defects for robotic-driven maintenance and prefabrication using residual attention U-Net. Autom. Constr..

[23] Atha D.J., Jahanshahi M.R. (2018). Evaluation of deep learning approaches based on convolutional neural networks for corrosion detection. Struct. Health Monit..

[24] Liu Y., Yao J., Lu X., Xie R., Li L. (2019). DeepCrack: A Deep Hierarchical Feature Learning Architecture for Crack Segmentation. Neurocomputing.

[25] Lee C.-Y., Xie S., Gallagher P., Zhang Z., Tu Z. Deeply-supervised nets. Proceedings of the Artificial Intelligence and Statistics.

[26] Hou Q., Zhang L., Cheng M.M., Feng J. Strip Pooling: Rethinking Spatial Pooling for Scene Parsing. Proceedings of the 2020 IEEE Conference on Computer Vision and Pattern Recognition (CVPR).

[27] Katsamenis I., Protopapadakis E., Bakalos N., Doulamis A., Doulamis N., Voulodimos A. (2023). A Few-Shot Attention Recurrent Residual U-Net for Crack Segmentation. arXiv.

[28] Vaswani A., Shazeer N., Parmar N., Uszkoreit J., Jones L., Gomez A.N., Kaiser Ł., Polosukhin I. (2017). Attention Is All You Need. Adv. Neural Inf. Process. Syst..

[29] Khan S., Naseer M., Hayat M., Zamir S.W., Khan F.S., Shah M. (2022). Transformers in Vision: A Survey. ACM Comput. Surv. (CSUR).

[30] Dosovitskiy A., Beyer L., Kolesnikov A., Weissenborn D., Zhai X., Unterthiner T., Dehghani M., Minderer M., Heigold G., Gelly S. (2020). An Image is Worth 16x16 Words: Transformers for Image Recognition at Scale. arXiv.

[31] Shao R., Shi Z., Yi J., Chen P.-Y., Hsieh C.-J. (2021). On the Adversarial Robustness of Vision Transformers. arXiv.

[32] Chen J., Lu Y., Yu Q., Luo X., Adeli E., Wang Y., Lu L., Yuille A.L., Zhou Y. (2021). Transunet: Transformers Make Strong Encoders for Medical Image Segmentation. arXiv.

[33] Zhang Y., Zhang L. (2023). Detection of Pavement Cracks by Deep Learning Models of Transformer and UNet. arXiv.

[34] Wu H., Chen S., Chen G., Wang W., Lei B., Wen Z. (2022). FAT-Net: Feature adaptive transformers for automated skin lesion segmentation. Med. Image Anal..

[35] Ali R., Chuah J.H., Talip M.S.A., Mokhtar N., Shoaib M.A. Crack Segmentation Network using Tversky Loss Function with Variable Alpha and Beta. Proceedings of the 2022 IEEE Symposium on Industrial Electronics & Applications (ISIEA).

[36] Jadon S. A Survey of Loss Functions for Semantic Segmentation. Proceedings of the 2020 IEEE Conference on Computational Intelligence in Bioinformatics and Computational Biology (CIBCB).

[37] Yi-de M., Qing L., Zhi-bai Q. Automated image segmentation using improved PCNN model based on cross-entropy. Proceedings of the 2004 International Symposium on Intelligent Multimedia, Video and Speech Processing.

[38] Nguyen Q.D., Thai H.T. (2023). Crack segmentation of imbalanced data: The role of loss functions. Eng. Struct..

[39] Fang J.F., Qu B., Yuan Y. (2021). Distribution Equalization Learning Mechanism for Road Crack Detection. Neurocomputing.

[40] Yeung M., Sala E., Schönlieb C.B., Rundo L. (2021). Unified focal loss: Generalising dice and cross entropy-based losses to handle class imbalanced medical image segmentation. Comput. Med. Imaging Graph..

[41] Taghanaki S.A., Zheng Y., Zhou S.K., Georgescu B., Sharma P., Xu D., Comaniciu D., Hamarneh G. (2019). Combo loss: Handling input and output imbalance in multi-organ segmentation. Comput. Med Imaging Graph..

[42] He K., Zhang X., Ren S., Sun J. Deep residual learning for image recognition. Proceedings of the 2016 IEEE Conference on Computer Vision and Pattern Recognition (CVPR).

[43] Yang F., Zhang L., Yu S., Prokhorov D., Mei X., Ling H. (2019). Feature Pyramid and Hierarchical Boosting Network for Pavement Crack Detection. arXiv.

[44] Kulkarni S., Singh S., Balakrishnan D., Sharma S., Devunuri S., Korlapati S.C.R. (2022). CrackSeg9k: A collection and benchmark for crack segmentation datasets and frameworks. Proceedings of the European Conference on Computer Vision.

[45] Zhou S., Song W. (2020). Deep learning-based roadway crack classification using laser-scanned range images: A comparative study on hyperparameter selection. Autom. Constr..

